# Emerging Technologies in Endoscopy for Gastrointestinal Neoplasms: A Comprehensive Overview

**DOI:** 10.7759/cureus.62946

**Published:** 2024-06-23

**Authors:** Gurkamal Singh Nijjar, Smriti Kaur Aulakh, Rajinderpal Singh, Sohbat Kaur Chandi

**Affiliations:** 1 Internal Medicine, Government Medical College, Amritsar, IND; 2 Internal Medicine, Sri Guru Ram Das University of Health Science and Research, Amritsar, IND

**Keywords:** diagnostic accuracy, minimally invasive, endoscopic techniques, artificial intelligence, gastrointestinal (gi) neoplasms

## Abstract

Gastrointestinal neoplasms are a growing global health concern, requiring prompt identification and treatment. Endoscopic procedures have revolutionized the detection and treatment of gastrointestinal tumors by providing accurate, minimally invasive methods. Early-stage malignancies can be treated with endoscopic excision, leading to improved outcomes and increased survival rates. Precancerous lesions, like adenomatous polyps, can be prevented by removing them, reducing cancer occurrence and death rates. Advanced techniques like chromoendoscopy, narrow-band imaging, and confocal laser endomicroscopy improve the ability to see the mucosa surface and diagnose conditions. Artificial Intelligence (AI) applications in endoscopy can enhance diagnostic accuracy and predict histology outcomes. However, challenges remain in accurately defining lesions and ensuring precise diagnosis and treatment selection. Molecular imaging approaches and therapeutic modalities like photodynamic therapy and endoscopic ultrasonography-guided therapies hold potential but require further study and clinical confirmation. This study examines the future prospects and obstacles in endoscopic procedures for the timely identification and treatment of gastrointestinal cancers. The focus is on developing technology, limits, and prospective effects on clinical practice.

## Introduction and background

Gastrointestinal (GI) neoplasms represent a significant global health burden, with a rising incidence and substantial morbidity and mortality rates. Global survival and mortality rates of different types of GI cancers vary widely based on factors such as cancer type, stage at diagnosis, region, and availability of medical resources. The five-year survival rate for pancreatic cancer is approximately 10% globally. Though early-stage pancreatic cancer has a high survival rate, it is rarely detected early and has aggressive progression. Likewise, mortality related to esophageal cancer is high (up to 80%), with most patients diagnosed at an advanced stage leading to poor prognosis. Five-year survival rates for stomach cancer are 31% globally, with higher survival rates in countries with established screening programs like Japan and South Korea. Survival rates are generally higher in high-income countries with better access to healthcare and advanced diagnostic techniques. Inappropriate biopsies and treatments for GI cancers not only pose significant health risks to patients, including unnecessary complications and adverse effects, but also lead to excessive costs that burden both the healthcare system and individuals. Thus, early detection and targeted treatment of malignant GI neoplasms are paramount in improving patient outcomes, reducing cancer-related morbidity, and enhancing overall survival rates [1]. Endoscopic techniques have revolutionized the diagnosis and treatment of GI tumors, offering minimally invasive approaches with high diagnostic accuracy and therapeutic efficacy [2].

Early detection of GI neoplasms such as pancreatic, esophageal, small intestine, gastric, colorectal, and liver cancers, is crucial for several reasons. Firstly, early-stage GI cancers are often asymptomatic or present with nonspecific symptoms, making them challenging to diagnose clinically [1]. As a result, many patients are diagnosed at advanced stages, when treatment options are limited, and prognosis is poor. Secondly, early detection allows for curative-intent treatment, such as endoscopic resection, which is associated with better outcomes and higher survival rates compared to advanced-stage disease [3]. For example, the five-year survival rate for early-stage esophageal cancer can exceed 90% with appropriate treatment, highlighting the importance of early detection strategies.

Furthermore, early detection of precancerous lesions, such as adenomatous polyps in the colon, can prevent the progression to invasive cancer through endoscopic polypectomy [4]. This not only reduces the incidence of colorectal cancer but also decreases cancer-related mortality rates. Thus, early detection strategies, including population-based screening programs and surveillance of high-risk individuals, are essential for reducing the burden of GI malignancies.

Endoscopic techniques have emerged as frontline modalities for the diagnosis, staging, and treatment of GI tumors [5]. Endoscopy provides direct visualization of the GI mucosa, enabling the detection of mucosal abnormalities, such as polyps, ulcers, and masses. In addition, advanced endoscopic imaging technologies, including chromoendoscopy, narrow-band imaging (NBI), and confocal laser endomicroscopy (CLE), enhance mucosal visualization and characterization, improving diagnostic accuracy. In this review, we explore the future directions and challenges in endoscopic techniques for the early detection and treatment of GI tumors, focusing on emerging technologies, limitations, and potential impacts on clinical practice.

## Review

Methods

A comprehensive literature search was conducted to gather relevant studies on emerging technologies in endoscopy for GI neoplasms. Databases including PubMed, MEDLINE, Embase, and Cochrane Library were systematically searched from January 2000 to December 2023. The search strategy employed a combination of keywords and Medical Subject Headings (MeSH) terms such as "endoscopy," "gastrointestinal neoplasms," "emerging technologies," "advanced imaging," "molecular imaging," "confocal laser endomicroscopy," "endoscopic ultrasound," and "radio-isotope detection." Boolean operators (AND, OR) were used to refine the search.

Studies were included if they focused on the application of advanced endoscopic technologies in the detection, diagnosis, or treatment of GI neoplasms, published in peer-reviewed journals, available in full text and written in English, and included human subjects. Two independent reviewers screened the titles and abstracts of the identified studies to determine their eligibility. Full-text articles of potentially relevant studies were then reviewed for inclusion. Disagreements were resolved through discussion or by consulting a third and fourth reviewer. Data extracted included study design, sample size, types of emerging endoscopic technologies evaluated, key findings, and clinical implications.

Artificial intelligence

AI has effectively been utilized in healthcare diagnostics, with a focus on acquiring knowledge and addressing issues by imitating human cognition. Machine learning (ML) and deep learning (DL) are particular categories of AI. ML primarily deals with statistical models and algorithms that have the ability to autonomously carry out intricate processes. DL, developed through artificial neural networks, specializes in deep neural networks, allowing for the creation of classification and recognition systems for focal images. Convolutional neural networks are the main DL algorithm for image recognition and processing.

Gastric Cancers

**Figure 1:**
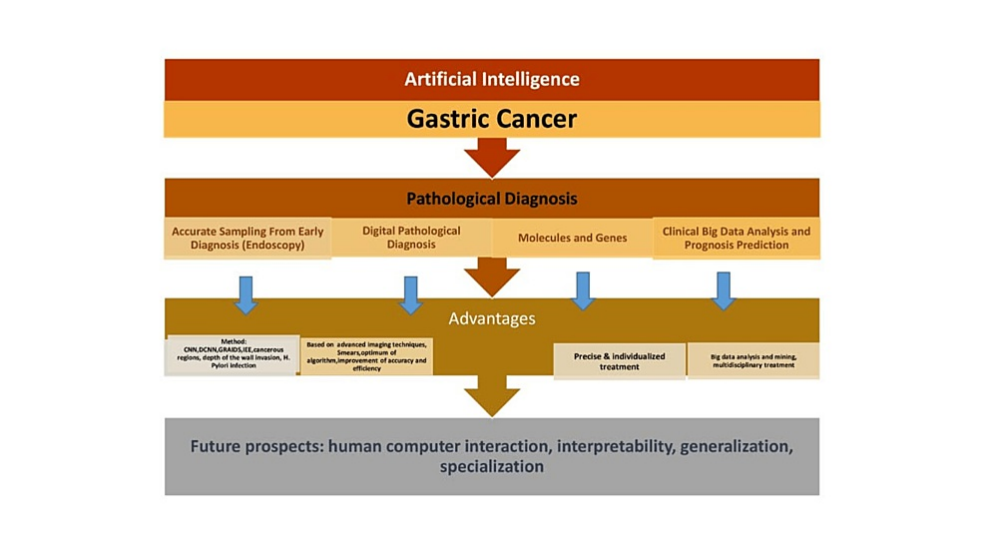
Application of artificial intelligence in gastrointestinal (GI) tumors. CNN: convolutional neural network, DCNN: deep convolutional neural network, GRAIDS: Gastrointestinal Artificial Intelligence Diagnostic System, IEE: image-enhanced endoscopy Image credits: Gurkamal Singh Nijjar

Endoscopists face challenges in detecting early gastric malignancies due to their occurrence within the context of gastric mucosal inflammation. A convolutional neural network (CNN) has been employed to identify gastric cancer (GC) in endoscopic imagery, achieving a sensitivity of 92.2% and a positive predictive value of 30.6% (Figure 1) [6]. Wu et al. developed a system utilizing a deep convolutional neural network (DCNN) to accurately identify early gastric cancer (EGC) without spots of limited visibility. The system demonstrated similar levels of sensitivity and specificity compared with all levels of endoscopists, reporting a positive predictive value of 91.3% and negative predictive value of 93.8% [7]. Luo et al. created a Gastrointestinal Artificial Intelligence Diagnostic System (GRAIDS), a system that may assist nonexpert endoscopists in diagnosing upper GI cancers at a level comparable to specialists [8]. Image-enhanced endoscopy (IEE) employs narrow-band spectrum or blue laser imaging to amplify the micro-vessel patterns and color disparities of the stomach mucosa and structural characteristics [9]. The diagnostic capability of magnifying-IEE (M-IEE) for GC has been proven, however, its expensive price and stringent restrictions restrict its widespread use [10]. The use of weak magnifying-IEE (WM-IEE) is widespread and cost-effective, making it a valuable tool for diagnosing high-risk lesions [11]. Magnifying NBI (ME-NBI) is an advanced optical device that provides precise and real-time diagnostic performance in EGC [12]. The computer-aided systems developed by CNN have demonstrated exceptional diagnostic accuracy, with an impressive area under the curve (AUC) of 99% [13]. Randomized controlled trials are essential for validating the diagnostic precision of artificial intelligence. In clinical practice, guidelines advocate for the utilization of multimodal light sources, including chromoendoscopy and white-light imaging (WLI) endoscopy. These guidelines highlight the significance of precise diagnosis and risk assessment in patients undergoing these procedures [14].

Distinguishing benign lesions (EGC) from advanced gastric cancer is challenging. Researchers have developed various systems to differentiate EGC from gastritis, including a CNN system that differentiates EGC from gastritis with high accuracy, sensitivity, and specificity. An AI-based system for GC and gastric ulcer classification was developed, with accuracies of 95.9% and 45.9% respectively [14]. An ultrasonic endoscopic (EUS) imaging-based CNN model achieved an 83.0% sensitivity, 75.5% specificity, and 79.2% accuracy in distinguishing gastrointestinal stromal tumors (GIST) from non-GISTs [15]. This has the potential to decrease the number of needless biopsies. EGC is categorized into two types: intramucosal and submucosal invasive tumors. The preferred treatment for EGC is endoscopic resection, which is favored for its low invasiveness and cost-effectiveness. Nevertheless, EUS does not have a significant impact on the initial assessment of tumor stage (T-staging) in patients with EGC [16]. As the demand for medical imaging grows, there is a requirement for AI systems that can provide more precise classification and improved accuracy. Scientists have created methods to enhance the diagnostic capability for EGC, incorporating an artificial intelligence classifier to distinguish between intramucosal and submucosal GC, as well as a diagnostic approach that involves collaboration between AI and endoscopists [17].

Colorectal Cancer

Colorectal cancer, the third most common cancer and second-most common cause of cancer-related deaths, is a significant issue. Colonoscopy is an essential procedure for screening and preventing the condition. It is capable of identifying and eliminating early signs of the disease, which reduces the likelihood of mortality by 67% and the occurrence of advanced cancer by 70% [18].

**Figure 2:**
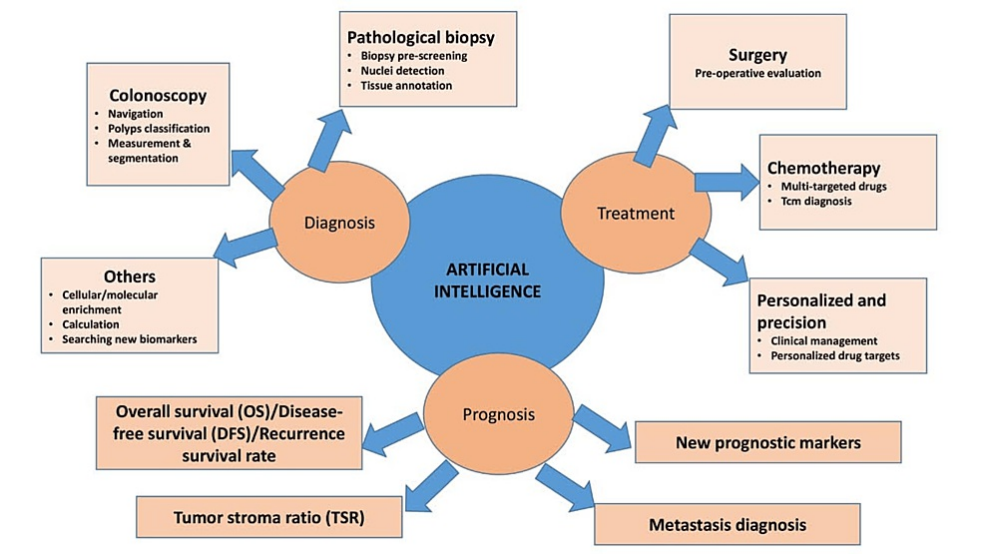
Application of artificial intelligence (AI) in colorectal cancer diagnosis and treatment. Image credits: Smriti Kaur Aulakh

The adenoma detection rate (ADR) refers to the percentage of colonoscopies in which at least one colorectal adenoma or adenocarcinoma is histologically discovered. ADR exhibits a negative correlation with the incidence of interval or post-colonoscopy colorectal cancer (PCCRC), which is expected to reach up to 3.5 per 1,000 individuals examined [19,20]. According to a study, for every 1.0% rise in ADR, there is a corresponding 3.0% drop in the risk of PCCRC and a 5% decrease in the risk of fatal interval colorectal cancer [19]. The adenoma missing rate (AMR) with WLI colonoscopy varies between 6% and 41%, depending on different polyps and operative factors [21]. AI can mitigate disparities in diagnostic proficiency among endoscopists resulting from variances in expertise, visual cognition, and other human elements. Several computer-aided diagnosis methods have been developed and employed in clinical settings to evaluate the benefits of improving ADR. The primary roles of AI in colorectal cancer screening are computer-aided detection (CADe) and computer-aided diagnosis or distinction (CADx) (Figure 2) [22]. CADe systems are designed to enhance ADR and identify adenomas by providing immediate visual information on polyps that were previously undetected [23].

Various studies have developed and applied computer-aided detection systems to improve colonoscopy efficiency and cost-effectiveness [24-26]. These systems have shown high accuracy and consistency in increasing polyp detection and reducing missed diagnoses of visible lesions. The application of CADe and colonoscopy indication were correlated with the ADR, and experience seemed to play a secondary role [24].

CADx tools are becoming more and more utilized for the histological prediction of colorectal polyps, with tissue biopsy being considered the most accurate method [14]. CADx utilizes endoscopic imagery to perform a qualitative diagnosis of colorectal cancers, demonstrating minimal difference among different observers [27]. The optical prediction of polyp histology is crucial in guiding subsequent therapy and plays a pivotal role in the "resect and discard" and "detect and leave" strategy [22]. Studies have demonstrated that surgically removing some colorectal neoplasms that are confined to the mucosa or superficial submucosa in one piece (known as en bloc R0 ER) can be considered a potentially curative treatment [28,29]. The effectiveness of CAD systems has been assessed by distinguishing between invasive and less invasive lesions [30]. Real-time image recognition systems have been employed to predict the histological diagnosis of colorectal lesions seen on NBI, with a 97.5% agreement between endoscopic diagnosis and CADx output [31]. Automated technologies that detect polyps in real-time can greatly decrease the occurrence of diagnostic errors and assist in making informed decisions about managing polyps [32]. However, AI systems are considered to be low-risk instruments that can assist, but not replace, the job of endoscopists. This makes it difficult to guarantee the added benefit of AI in clinical practice.

Advanced imaging modalities

The majority (90%) of esophageal malignancies are classified as esophageal squamous cell carcinoma (ESCC), with the highest incidence observed in East Asia, Iran, and Africa. Screening endoscopy and Lugol's chromoendoscopy are considered the most reliable methods in these regions, however they still have some limitations in terms of specificity [33]. Esophageal adenocarcinoma (EAC) is the most common subtype of esophageal cancer in Western countries. Population-based screening endoscopy is not advised due to the low occurrence rate. However, researchers are currently assessing other techniques like as ultrathin endoscope and Cytosponge, along with biomarkers [34].

Macroscopic Imaging Techniques

The most commonly used method for assessing gastrointestinal lesions is conventional WLE due to its distinct anatomy [33]. Nevertheless, there is a necessity to enhance the diagnostic efficacy of WLE. High-definition endoscopes have become readily accessible since the late 1990s, allowing for more detailed investigation of mucosal patterns and displacing standard-definition endoscopes as the preferred method [35]. The future advancement of high-definition WLE can be enhanced by making more advancements in the optical field of view (FOV) and resolution, along with the inclusion of 3D imaging capacity.

Virtual chromoendoscopy (VCE) utilizes the differences in how light interacts with tissue to emphasize the characteristics of the mucosal surface. The first version of VCE consists of NBI, Fuji Intelligent Chromo Endoscopy (FICE), and iScan. Additionally, more recent techniques, like blue laser imaging, have been implemented. NBI is the most commonly studied VCE modality, and it has shown promise in improving diagnostic yield for dysplasia/cancer detection in Barrett's esophagus and gastric precancerous histology [36]. VCE holds great potential for enhancing the visualization of GI lesions without extra cost or the use of external dyes. However, there is a need to establish and validate universal classification criteria and to study associated learning curves and interobserver reliability [33]. Multispectral or hyperspectral imaging has also demonstrated its usefulness in characterizing GI lesions by providing more spectral dimensions. Hyperspectral imaging, which provides real-time data, has been successfully incorporated into endoscopic systems. The early clinical evaluations of this technology in the gastrointestinal tract have been recorded [37]. It is anticipated that the detection of GI lesions will continue to enhance with the advancement of VCE technology and the standardization of clinical applications [38].

Chromoendoscopy with exogenous dyes is a method employed to enhance the distinction of mucosal characteristics, particularly in lesions that may seem inconspicuous or smooth during WLE. Clinically, two primary categories of dyes are utilized: absorptive dyes, such as methylene blue and Lugol's iodine, and contrast stains, such as indigo carmine. These dyes are both very sensitive and cost-effective, which makes them a suggested choice for monitoring of Barrett's esophagus [39-41].

Ultrathin endoscopes are specifically developed to access and examine smaller organs within the body's cavities. This enables endoscopic procedures to be carried out without the need for anesthesia, making it possible to do them in outpatient settings. However, they necessitate expertise in image analysis and have restricted pixel resolution and mechanical dexterity. Transnasal endoscopy, which utilizes ultrathin endoscopes, is being explored as a potential alternative for screening Barrett's esophagus (BE) [42]. However, its ability to effectively monitor BE is hindered by its relatively lower quality of imaging [43].

Capsule endoscopy, which was authorized by the Food and Drug Administration (FDA) in 2001, enables a convenient and secure inspection of the gastrointestinal system. This technology has greatly revolutionized the treatment of small bowel disorders [44]. Studies have demonstrated that it can effectively display significant anatomical features of the esophagus and the entire stomach [39]. However, it is not advisable to use it for screening Barrett's esophagus due to its moderate level of accuracy. Capsule endoscopy has been employed to examine larger sections of the GI tract, and recent clinical findings have demonstrated enhanced image quality and mobility [45]. Further investigations are necessary to evaluate its diagnostic efficacy. Hence, CE provides a secure and comparatively affordable option for improving mucosal contrast. However, it necessitates user proficiency and agreement on established criteria for interpretation.

Microscopic Imaging Modalities

Macroscopic imaging techniques serve as the basis for gastrointestinal endoscopy, nevertheless, microscopic examination is still considered the most reliable method for diagnosing malignancy. This can result in superfluous biopsies, increased healthcare expenses, diagnostic delays, and failure to continue monitoring [46]. Consequently, there has been a persistent need to create in vivo endoscopic methods that can aid in the identification and analysis of lesions with extremely detailed resolution. Endomicroscopic imaging techniques encompass several systems, including commercially accessible ones like CLE, volumetric laser endomicroscopy (VLE), as well as investigative research systems such as high-resolution microendoscope (HRME) and capsules based on optical coherence tomography (OCT) [46]. CLE has been utilized for the identification of dysplasia associated to Barrett's esophagus, gastric tumors, and colorectal cancer [47]. Endocytoscopy, like magnification WLE, provides reflectance imaging with optical magnification of up to 150 times [48]. However, it is specifically enhanced for the detailed examination of GI lesions at the cellular level. Recent investigations have demonstrated that commercial endocytoscopy equipment can accurately characterize GI lesions at the cellular level, demonstrating great diagnostic performance [49].

OCT is a high-resolution imaging technology that generates detailed images. The device has an axial resolution of around 10 µm and a lateral resolution of roughly 30 µm [50]. It is compatible with endoscopic imaging of hollow organs, such as the gastrointestinal tract and cardiovascular systems. Various configurations of OCT devices have been developed, including probe- and balloon-based OCT that can be maneuvered through an endoscope working channel, as well as a capsule-based design that is suitable for primary care settings [51].

The VLE, an OCT-based imaging system for esophageal imaging, was commercially available in 2013. The procedure utilizes a balloon catheter to position the OCT probe, allowing for consistent scanning of a 6 cm section of the esophagus with a 3 mm imaging depth in a time frame of 90 seconds [33]. Since its inception, studies have consistently demonstrated a diagnosis accuracy rate of 87% for dysplasia associated to Barrett's esophagus [52].

Endoscopic OCT has recently incorporated a capsule enclosure, which enables the inclusion of small optomechanical components at the probe's distal end [53]. This technological development improves the caliber and differentiation of visual representation. This allows for the utilization of high-speed OCT angiography to observe the microvasculature located beneath the surface of the gastrointestinal tract [54]. Moreover, a piezoelectric probe has been developed to enhance the ability to do forward-viewing OCT imaging of colorectal polyps [55].

The HRME is an affordable fiber-optic fluorescence microscope capable of capturing detailed images of nuclear structure at a level below the size of a cell [56]. It has demonstrated efficacy in detecting dysplasia associated to Barrett's esophagus and esophageal squamous cell carcinoma in the upper gastrointestinal tract [57]. Additional in vivo microscopy techniques, such as multiphoton endomicroscopy and spectroscopic probes, have been created to investigate the cellular structure and molecular components of GI abnormalities. These technologies require additional assessment in larger clinical trials [58].

Molecular Imaging

Molecular imaging is a developing discipline in the field of oncology that has the ability to identify GI lesions at an earlier stage than changes in their physical structure. The technique employs several molecular probes, including as antibodies (trastuzumab and bevacizumab), peptides (RGD peptides targeting integrins, particularly αvβ3 integrins involved in tumor angiogenesis and metastasis, and Bombesin which target gastrin-releasing peptide receptors), nanoparticles (iron oxide and gold nanoparticles due to their strong optical properties), aptamers (AS1411- a DNA aptamer that targets nucleolin, and aptamer based probes for epidermal growth factor receptor-EFGR), and affibodies (targeting HER2 and EFGR) [49]. Usually, these probes are labeled with fluorescent markers, radiotracers, and other detectable agents. These are utilized together with fluorescence imaging techniques such as CLE or widefield fluorescence endoscopes [59]. Recent research have shown that ex vivo and in vivo applications have the potential to be used in human trials [60]. These applications can overcome the constraints of many widefield endoscopic techniques and allow for safe longitudinal studies without the need for tissue removal. Subsequent investigations will prioritize the development of innovative probes and imaging equipment, with a tight emphasis on ensuring safety during their clinical application.

In addition to fluorescence imaging, radioisotope detection techniques offer another promising approach for early GI cancer detection. Positron emission tomography (PET) and single photon emission computed tomography (SPECT) are widely used molecular imaging modalities that utilize radiolabeled tracers to visualize metabolic and molecular processes in vivo [33]. These techniques employ radioisotopes such as fluorodeoxyglucose (FDG) in PET, which accumulates in cancerous tissues due to their high metabolic activity, allowing for precise localization and characterization of tumors. The integration of PET with computed tomography (PET/CT) enhances the anatomical resolution and provides comprehensive data on the spatial distribution of lesions. SPECT, on the other hand, utilizes gamma-emitting radioisotopes like technetium-99m, and has shown efficacy in detecting certain GI malignancies and their metastases [35]. The ability to quantify tracer uptake in real-time offers a significant advantage in monitoring treatment response and disease progression. As molecular imaging advances, the development of novel radiotracers targeting specific cancer biomarkers will further improve the sensitivity and specificity of these techniques, potentially leading to earlier diagnosis and better patient outcomes in GI cancers.

Therapeutic modalities

Endoscopic Mucosal Resection

**Figure 3:**
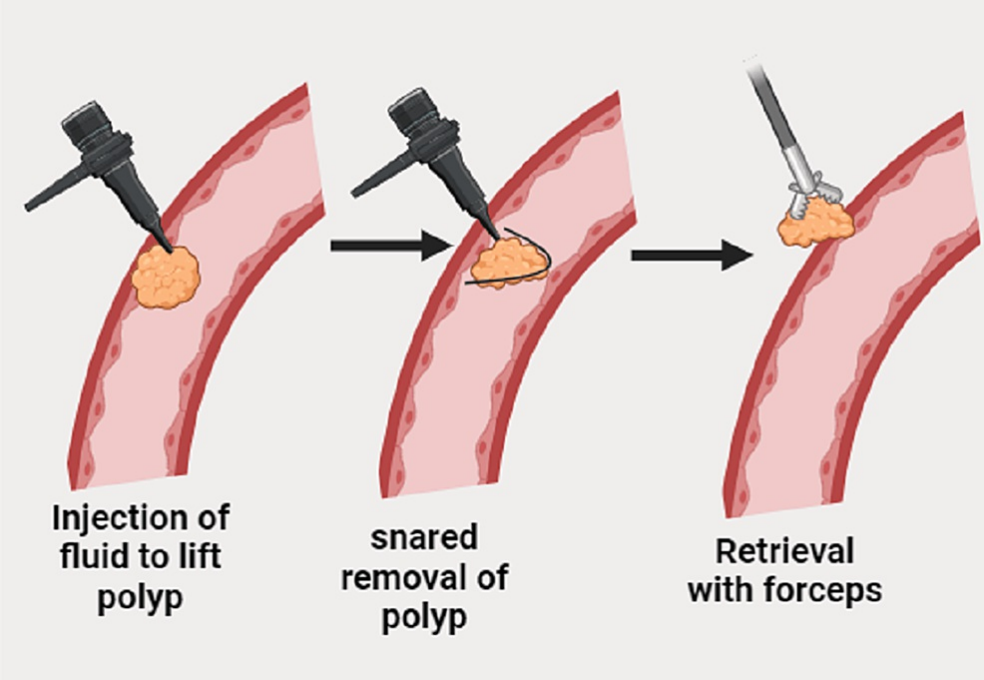
Endoscopic mucosal resection (EMR) technique. Image credits: Sohbat Kaur Chandi

The initial implementation of the endoscopic mucosal resection (EMR) method took place in Japan in 1984, and subsequently, multiple approaches have been devised. The EMR technique, with a cap-fitted panendoscope, was created in 1992 specifically for removing superficial esophageal cancer (SEC) [61]. This procedure can also be directly used for removing EGC (Figure 3). This method entails attaching a clear plastic cap to the end of a regular endoscope [62]. Inside the cap, there is a snare that is already formed into a loop and positioned along the inner groove of the cap's distal half. This configuration enables the operator to excise lesions that are drawn into the cap using suction. This procedure allows for the safe removal of intramucosal tumors that are 2 cm or smaller in diameter [61].

Another technique for EMR, initially involving ligation and later expanded to include multiband ligation, utilizes band ligation to form a "pseudopolyp" by drawing the lesion into the banding cap and placing a band underneath it [63]. There exists a diverse range of EMR techniques employed in clinical environments, nevertheless, the fundamental steps involved in executing this operation are as follows: The procedure involves four steps: (1) defining the side boundary, with or without chromoendoscopy, (2) marking the area with a short burst of electrocautery or argon plasma coagulation, (3) injecting a substance under the mucosa to elevate the lesion, and (4) removing the lesion.

Prior to the advent of endoscopic submucosal dissection (ESD), the most efficient technique for removing bigger tumors in a single piece was circumferential precutting using endoscopic mucosal resection (EMR-P) [64]. Electromagnetic radiation therapy (EMR-P) can be considered as a viable option for treating intramucosal malignancies that are smaller than 2 cm, as an alternative to ESD [62]. Nevertheless, tumors that are larger than 2 cm in size cannot be entirely removed using EMR in a single procedure [65]. Furthermore, fragmented removal, known as piecemeal resection, may raise the likelihood of local recurrence and result in insufficient histologic staging.

Endoscopic Submucosal Dissection

ESD, or endoscopic submucosal dissection, is a minimally invasive procedure that was designed in the mid-1990s in Japan with the purpose of removing early stage gastrointestinal cancers [66]. An electrosurgical knife is used in this endoscopic resection procedure to precisely dissect the tissue above the muscularis propria [67].

A study found that patients receiving ESD had significantly higher rates of en bloc resection and curative resection compared to those who underwent EMR [68]. In addition, ESD was linked to a reduced risk of local recurrence. There was no discernible disparity in the risk of bleeding between ESD and EMR. There has been a rise in the number of practitioners outside of Asia who are skilled and experienced in performing ESD. ESD is now widely regarded as the preferred endoscopic treatment for patients with EGC, as stated in specific published recommendations [69]. Magnifying endoscopy with narrow-band imaging and ESD (MAG-ESD) is another highly effective technique for the treatment of early gastric cancer, combining precise imaging to identify lesions with minimally invasive removal. This approach enhances the accuracy of tumor delineation and ensures complete resection, significantly improving patient outcomes [29].

Radiofrequency Ablation

Radiofrequency ablation (RFA) has been investigated in small clinical studies as a potential therapy for treating gastric low grade intraepithelial neoplasia (LGIN) [70,71]. RFA offers several advantages, including a straightforward procedure, reduced risk, cheaper cost, and quick recovery. Nevertheless, the effectiveness of it and particularly the prognosis risk factors remain incompletely comprehended. The underlying mechanism of RFA involves inducing the motion of electrically charged particles within tissues using high-frequency alternating current [71]. This generates heat, leading to the evaporation, desiccation, contraction, and detachment of water present inside and outside cells. Consequently, this process results in aseptic necrosis. The power output and energy density of each RFA are fixed and do not change as the process continues. RFA is a straightforward procedure that can be successfully carried out by an endoscopic physician with the necessary skills to operate the gastroscope. Simultaneously, there are no occurrences of bleeding, perforation, infection, or any other significant problems following RFA. The aforementioned benefits indicate that RFA has promising potential for clinical development.

During RFA, it is crucial for the electrode to be firmly adhered to the mucosal surface in order to ensure optimal transmission of energy. Prior to the subsequent ablation procedure, it is imperative to completely eliminate the necrotic mucosal tissue present on the lesion surface from the prior ablation. This is necessary to prevent any decrease in energy conduction and to guarantee the desired ablation outcome [70]. The aforementioned procedures can be accomplished by twisting the endoscope, inhaling, and aerating. The RFA method has multiple advantages. Furthermore, apart from the study's positive results in terms of effectiveness and safety [72], the technique is straightforward and can be easily acquired, typically requiring 10-20 minutes to be fully performed. Furthermore, RFA offers a cost-effective solution, hence alleviating the financial strain on patients. Furthermore, patients are able to consume food the day following the surgical procedure, without the necessity of prophylactic antibiotics, which promotes the process of recuperation. Lastly, RFA can be conducted as an outpatient procedure, eliminating the need for hospitalization and thereby conserving valuable medical resources. Hence, considering the aforementioned benefits, it is worthwhile to investigate the future clinical use and widespread adoption of RFA. The primary risk factors for the recurrence of low-grade LGIN after RFA include H. pylori infection and a clinical course lasting more than one year. Secondary RFA therapy may be undertaken for cases that have relapse or recurrence.

A study evaluate the safety and technical success of endoscopic RFA for palliative treatment of malignant hilar bile duct obstruction [73]. This study aimed to evaluate the impact of electrosurgical factors on the size of the necrotic region caused by a newly approved endoscopic RFA catheter, using an ex-vivo pig liver model. Afterwards, a review was performed on all patients who underwent endoscopic RFA for malignant biliary blockage. After RFA, all patients had the placement of an extra plastic stent in the biliary tree. The study determined that Endoscopic RFA is a straightforward and technically proficient method. Nevertheless, hemobilia, which may be linked to RFA, was observed in three of our patients. Thus, it is necessary to conduct more extensive prospective studies in order to thoroughly assess the safety and effectiveness of this highly promising new approach.

Photodynamic Therapy

Photodynamic therapy (PDT) is occasionally employed in GIC due to its ability to specifically target tumor tissue, while causing minimum harm to nearby healthy tissues, having few systemic side effects, and allowing for repeated procedures. PDT has been utilized in the treatment of various types of cancer, including esophageal cancer, gastric cancer, bile duct cancer, and colorectal cancer [74]. Nevertheless, the effectiveness of PDT in a clinical setting is also impacted by several factors, with the primary considerations being the photosensitizer, the light source used for irradiation, and the skill of the operators. Photosensitizer, excitation light source, and tissue oxygen are essential components in PDT. The effective integration of these three elements is crucial for achieving anti-tumor effects. However, the process of combining them is a difficult matter. Determining the various parameters such as optimal dosage of photosensitizer for a particular tumor, type of light to be used, irradiation using specific wavelength, energy, output mode, the duration of the irradiation process, continuous or interval scale, and the duration between two instances of irradiation, are crucial for the operator to proceed to develop the treatment plan. These factors will impact the effectiveness of PDT, however, these effects are widely recognized. During the clinical treatment, He et al. discovered that patients exhibit varying degrees of responsiveness to PDT [48]. Furthermore, even the same patients may experience variable clinical outcomes when receiving treatment at different time intervals. Thus, clinicians should examine the elements that influence PDT including the patient's individual conditions, tumor features, and tumor microenvironment. This is crucial for enhancing the therapeutic efficacy of PDT.

Nishie et al. explained that PDT utilizes the interaction between a photosensitizer and light to produce reactive oxygen species, such as singlet oxygen, which have the potential to be therapeutically effective in cancer cells [75]. Several sugar-conjugated chlorins have been shown to exhibit more potent anticancer effects in PDT compared to talaporfin sodium (TS), a second-generation photosensitizer being utilized in Japan. The researchers in this study created a new compound called glucose-conjugated chlorin e6 (G-chlorin e6) and examined its ability to inhibit tumor growth. G-chlorin e6 had exceptional tumor specificity, and PDT utilizing G-chlorin e6 exhibited the most potent anti-tumor effects compared to all sugar-conjugated chlorins investigated in our study. G-chlorin e6 is widely regarded as the most effective photosensitizer for the advancement of PDT in the future.

Endoscopic Ultrasound-Guided Interventions

EUS allows for the imaging of malignancies, such as GIST, and the collection of samples using a fine-needle aspiration (FNA) technique for cytology analysis [76]. Mass spectrometry has been demonstrated to aid in the difficult evaluation of cystic pancreatic lesions, which are possible precursors of pancreatic adenocarcinoma [77]. However, in GISTs, samples obtained using endoscopic ultrasound-guided fine-needle aspiration (EUS-FNA) typically do not provide a definitive diagnosis [76,78]. This also results in a clear absence of prognostic information derived from the tumor's mutation profile and proliferation rate. This limitation of EUS-FNA poses a significant barrier to the prompt customized treatment of individuals with GIST. Faced with the challenges in characterizing GISTs, clinicians must make a decision regarding surgical resection solely on the suspicion of malignancy, without any information about the tumor's growth rate. Ultimately, the determination regarding the costly neoadjuvant imatinib therapy can solely rely on probability rather than the specific mutation profile of KIT and platelet derived growth factor alpha (PDGFRA). The main objective of the Hedenstrom study was to assess the use of EUS-guided sampling for diagnosing and characterizing GIST [79]. The study determined that EUS-guided biopsy sampling is precise for the initial diagnosis and characterisation of GISTs and enables the anticipation and assessment of tumor response to neoadjuvant imatinib therapy. A second study by the same set of researchers investigated the diagnostic accuracy and clinical significance of using EUS-guided biopsy sampling (EUS-FNB) with a reverse bevel needle, as opposed to EUS-FNA, in the evaluation of subepithelial lesions (SEL) [80]. The study concluded that using a reverse bevel needle in EUS-FNB is both safe and more effective than EUS-FNA for accurately diagnosing subepithelial lesions. This biopsy sample method enables a logical clinical approach and precise treatment.

Another set of researchers conducted a study to evaluate the accuracy and the cytologic features of EUS-guided paracentesis in the diagnosis and staging of malignant neoplasms [81]. All EUS-guided paracenteses of ascitic fluid performed retrospectively in 101 patients. Corresponding EUS findings, cytology and histology slides, and follow-up information were reviewed. EUS-guided paracentesis is a valuable aid in the cytologic diagnosis of malignant ascites. It is particularly useful when no abnormality is identified by CT

Addressing limitations and barriers

Accurate localization and navigation remain challenging, especially for small and flat lesions or those located in difficult-to-access areas of the gastrointestinal tract. Improvements in endoscopic imaging and navigation systems are necessary to overcome these challenges. Additionally, endoscopic techniques for gastrointestinal tumor detection and treatment require specialized training and expertise. Standardized training programs and simulation-based training can help improve the skills of endoscopists, reducing variability in outcomes. Furthermore, the adoption of advanced endoscopic technologies can be hindered by high costs and limited accessibility, particularly in resource-limited settings. Efforts to reduce equipment costs, increase training opportunities, and promote technology transfer are essential for widespread adoption. Despite its minimally invasive nature, endoscopic treatment is associated with risks such as bleeding, perforation, and post-procedural strictures. Strategies to mitigate these risks, such as improved patient selection criteria and the development of safer techniques, are crucial for ensuring patient safety.

## Conclusions

Timely identification of gastrointestinal neoplasms is crucial because they frequently lack symptoms during the initial stages. Endoscopic excision of early-stage malignancies results in superior outcomes and increased rates of survival. Identifying precancerous lesions such as adenomatous polyps enables the prevention of cancer by removing the polyps, which in turn reduces the occurrence and death rates associated with cancer.

Advanced techniques in endoscopy, such as chromoendoscopy, NBI, and CLE, improve the ability to see the surface of the mucosa and accurately diagnose conditions. VCE shows potential for enhancing lesion detection without incurring extra expenses or using external dyes, while there is a requirement for uniform categorization criteria. EMR, ESD, and RFA provide minimally invasive therapeutic alternatives. Nevertheless, there are still difficulties in accurately defining lesions and assuring precise diagnosis and selection of treatment. AI applications in endoscopy have the potential to enhance diagnostic accuracy, assist in lesion classification, and predict histology outcomes.

Further study and clinical validation are needed for molecular imaging approaches and therapeutic modalities such as PDT and EUS-guided therapies, despite their promising potential. It is crucial to address the challenges in achieving precise localization, navigation, and accessibility of sophisticated endoscopic technology. Implementing standardized training programs, implementing cost reduction measures, and developing techniques to limit procedural risks are essential for promoting widespread adoption and enhancing patient outcomes in the management of GI neoplasms.
